# A standalone incompatible insect technique enables mosquito suppression in the urban subtropics

**DOI:** 10.1038/s42003-022-04332-6

**Published:** 2022-12-27

**Authors:** Qin Zeng, Lingzhi She, Hao Yuan, Yuying Luo, Renke Wang, Wei Mao, Weifeng Wang, Yueting She, Chaojun Wang, Mengyi Shi, Ting Cao, Renxian Gan, Yongjun Li, Jiayi Zhou, Wei Qian, Shixiong Hu, Yong Wang, Xiaoying Zheng, Kuibiao Li, Lianyang Bai, Xiaoling Pan, Zhiyong Xi

**Affiliations:** 1 The Key Laboratory of Model Animals and Stem Cell Biology in Hunan Province, Department of Medical Laboratory Science, Hunan Normal University School of Medicine, Changsha, Hunan PR China; 2 The Key Laboratory of Protein Chemistry and Developmental Biology of Fish of Ministry of Education, Hunan Normal University, Changsha, Hunan PR China; 3 Guangzhou Wolbaki Biotech Co., Ltd, Guangzhou, Guangdong PR China; 4 Hunan Provincial Center for Disease Control and Prevention, Changsha, Hunan PR China; 5 Department of Forensic Science, School of Basic Medical Science, Central South University, Changsha, Hunan PR China; 6 Sun Yat-sen University—Michigan State University Joint Center of Vector Control for Tropical Diseases, Zhongshan School of Medicine, Sun Yat-Sen University, Guangzhou, Guangdong PR China; 7 Guangzhou Center for Disease Control and Prevention, Guangzhou, Guangdong PR China; 8 Hunan Academy of Agricultural Sciences, Changsha, Hunan PR China; 9 Department of Microbiology and Molecular Genetics, Michigan State University, East Lansing, MI USA; 10 Present Address: Department of Pathogen Biology, School of Medicine, Jinan University, Guangzhou, PR China

**Keywords:** Applied microbiology, Translational research, Microbiology techniques

## Abstract

The strong suppression of *Aedes albopictus* on two Guangzhou islands in China has been successfully achieved by releasing males with an artificial triple-*Wolbachia* infection. However, it requires the use of radiation to sterilize residual females to prevent population replacement. To develop a highly effective tool for dengue control, we tested a standalone incompatible insect technique (IIT) to control *A. albopictus* in the urban area of Changsha, an inland city where dengue recently emerged. Male mosquitoes were produced in a mass rearing facility in Guangzhou and transported over 670 km under low temperature to the release site. After a once-per-week release with high numbers of males (phase I) and a subsequent twice-per-week release with low numbers of males (phase II), the average numbers of hatched eggs and female adults collected weekly per trap were reduced by 97% and 85%, respectively. The population suppression caused a 94% decrease in mosquito biting at the release site compared to the control site. Remarkably, this strong suppression was achieved using only 28% of the number of males released in a previous trial. Despite the lack of irradiation to sterilize residual females, no triple-infected mosquitoes were detected in the field post release based on the monitoring of adult and larval *A. albopictus* populations for two years, indicating that population replacement was prevented. Our results support the feasibility of implementing a standalone IIT for dengue control in urban areas.

## Abstract (web summary)

A field trial in Changsha, China, involving the release of a triple *Wolbachia*-infected strain of the mosquito, *Aedes albopictus*, demonstrates the feasibility of using an incompatible insect technique to suppress disease vector mosquito populations in urban subtropic environments.

## Introduction

Dengue, a widespread mosquito-borne infectious disease, is caused by the transmission of dengue virus between humans and mosquito vectors, *Aedes aegypti* and *Aedes albopictus*^1^. Approximately 50-100 million infections occur annually in over 100 endemic countries with almost half of the world’s population at risk^2^. In recent years, dengue virus transmission has increased predominantly in urban and semiurban areas, which has led to major international public health concerns^3^. However, no specific antiviral drug has been approved for dengue^1^. Due to the challenge in developing effective immunity against all four antigenically different dengue serotypes simultaneously to avoid antibody-dependent enhancement, only one licensed vaccine is currently available to those 9–45 years of age who have been previously infected^4^. Accordingly, vector control is the primary intervention for dengue control and prevention. During the last two decades, prominent progress has been made in developing a vector control tool based on *Wolbachia*.

*Wolbachia pipientis*, a maternally inherited gram-negative bacterium, is a diverse, ubiquitous endosymbiont of arthropods^5^. It can manipulate mosquito reproduction through cytoplasmic incompatibility (CI)^6^, a phenomenon of conditional embryonic lethality that results from mating between a *Wolbachia*-infected male and a female that is either uninfected or infected by a different *Wolbachia* strain. CI offers the theoretical basis for the incompatible insect technique (IIT), in which inundative release of *Wolbachia*-infected males is used to induce sterile matings with wild-type females in the field, resulting in strong population suppression^7–11^. Given that some *Wolbachia* strains can induce pathogen blocking in mosquitoes, another *Wolbachia*-based vector control strategy referred to as population replacement involves the release of infected females to utilize CI for spreading *Wolbachia* into the target population to reduce the mosquito’s ability to transmit dengue virus due to the advantage of infected females in reproduction compared to their uninfected counterparts. Both population replacement and IIT strategies require the establishment of a novel strain of *Wolbachia* infection (or transinfection) in the targeted species that has perfect maternal transmission and can induce CI towards the field population. Multiple *Wolbachia* strains have been introduced to both *A. aegypti* (without native *Wolbachia* infection) and *A. albopictus* (with a native superinfection of *w*AlbA and *w*AlbB) to develop *Wolbachia* for dengue control. Due to the lack of a perfect sex separation technique, however, the operation of IIT demands avoiding population replacement caused by accidental release of fertile females as mating between females and males carrying the same *Wolbachia* is compatible, resulting in failure in further population suppression. Currently, large-scale implementation of IIT uses either radiation or artificial intelligence to prevent the risk of population replacement. Notably, IIT combined with the irradiation-based sterile insect technique (SIT) has achieved strong suppression of *A. albopictus* populations on the islands of Guangdong Province, China^11,12^, as well as *A. aegypti* populations in Singapore^13^, Mexico^14^ and Thailand^15^. The high efficacy of IIT to suppress *A. aegypti* has also been accomplished in the U. S.^9^, northern Australia^7^ and Singapore^13^ through the use of artificial intelligence to augment the efficient sex sorting of male mosquitoes. Although both radiation and artificial intelligence increase the cost of the IIT program, these repeatable successes demonstrate the feasibility of area-wide application of *Wolbachia*-based IIT for the suppression of dengue vector mosquitoes. Due to the geographical expansion of dengue from southeastern coastal areas to inland areas of China in recent years^16^, there is an urgent need for highly effective mosquito-control tools, particularly against *A. albopictus*, the dominant *Aedes* mosquito species and the main vector of dengue in urban areas^17^.

The Chinese inland cities have longer and colder winters than southeastern coastal areas, such as Guangzhou, which strongly limits the year-round activities of the *A. albopictus* population. Mosquito eggs undergo quiescence during periods with unfavorable environmental conditions, such as winter and the dry season. *Wolbachia* transinfection has been observed to negatively impact egg development and viability over time by reducing resistance to desiccation^18^. The *Wolbachia* density in the transinfected *A. aegypti* also decreased over storage time, with emergent infected females showing fertility loss after egg quiescence^19^. Moreover, the high fitness costs associated with *Wolbachia* infection under real-world conditions have been reported to prevent population replacement in the field, such as failure in the sustainable establishment of *w*MelPop and *w*Mel in Vietnam^20,21^. In particular, the increased susceptibility of *w*Mel-infected *A. aegypti* to high temperatures and probably other environmental factors resulted in *Wolbachia* being removed from the field population in both central and northern Tri Nguyen four years after release^21^. Low temperatures also suppress the density of *w*AlbB and *w*Mel in transinfected *A. aegypti*, although native *Wolbachia* infection can be maintained in *A. albopictus* due to its high cold tolerance^22^. The adverse impact of extended winters and low temperatures on *Wolbachia* is likely to increase the invasion threshold for *Wolbachia* to be established and sustained across mosquito seasons in the field, raising the possibility of effectively suppressing the population by IIT with population replacement prevented even at our current sex sorting accuracy^23^ without further irradiation. With much emphasis on the risk of population replacement to IIT, very little attention has been paid to the tolerance of the field population to *Wolbachia* introduced by the escaped females during population suppression, which calls for a full evaluation of the feasibility and effectiveness of a standalone IIT for dengue control in subtropical monsoon climate areas.

Herein, we report the application of a standalone IIT using an *A. albopictus* HC line with triple infection of *w*Pip, *w*AlbA, and *w*AlbB in Furong District of Changsha city, an inland city that exhibited the emergence of dengue cases in 2019^11,24^. The HC males, which share the same haplotype H1 with the local *A. albopictus* population based on phylogenomic analyses, were produced through the upgraded automatic mosquito pupae sex sorter without further irradiation and were transported over 600 km to the release site under low temperature. Subsequently, the release of HC males twice per week resulted in strong suppression of the *A. albopictus* population for 14 weeks, and the suppression effect was sustained during the post-release period. Importantly, no evidence of population replacement was observed in the field based on monitoring of the population over two years.

## Results

### Selection of field sites in urban areas for standalone application of IIT for mosquito suppression

**Fig. 1:**
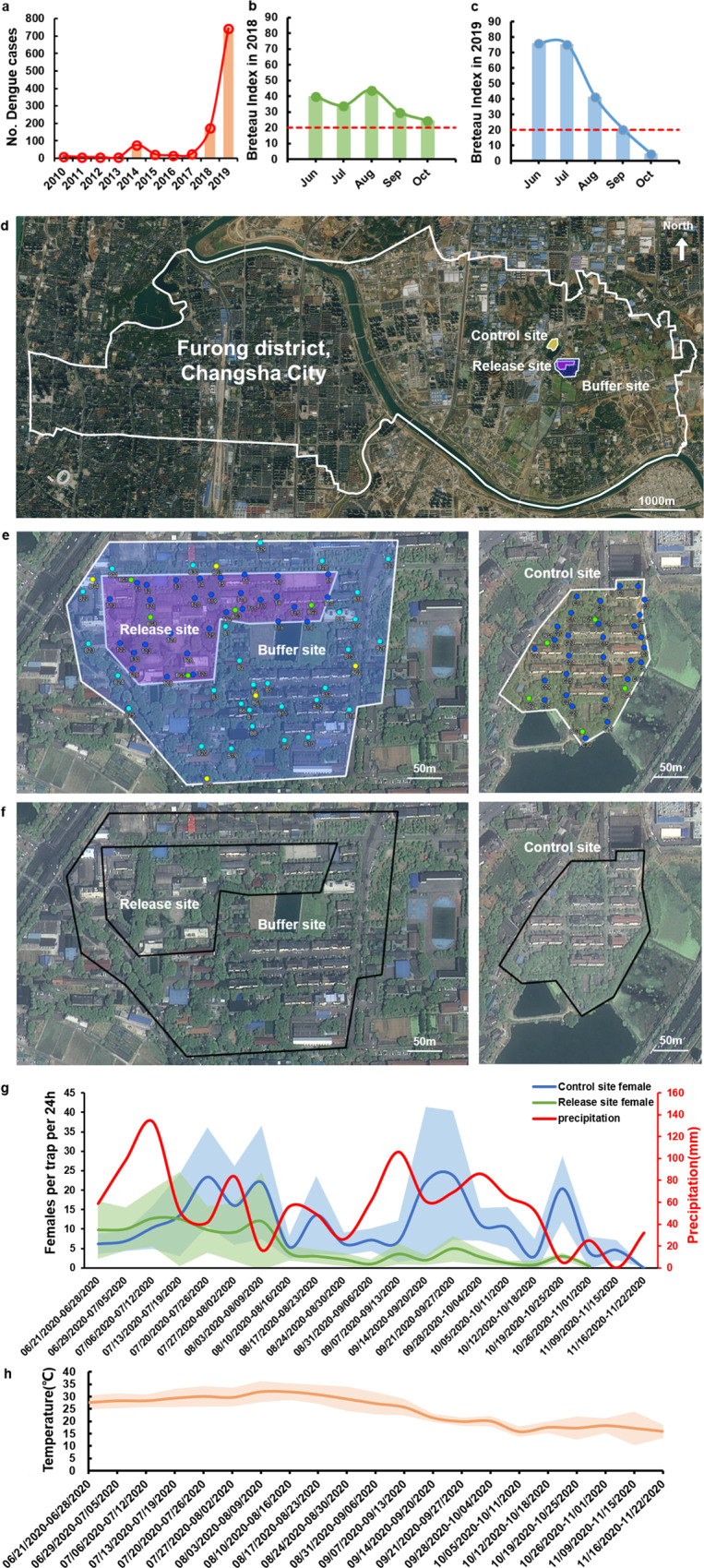
Field sites, mosquito baseline density, and weather data. **a** Annual number of dengue cases reported in Hunan Province from 2010 to 2019. Breteau Index in Changsha, Hunan in 2018 (**b**) and 2019 (**c**) provided by the Hunan CDC. The red dotted line represents the predicted Breteau Index threshold level of 20 with a high risk for dengue transmission according to the guidelines from the China CDC. The data of dengue cases reported in Hunan Province from 2010-2019 and *Aedes* data were obtained from the Chinese National Notifiable Infectious Disease Reporting Information System (CNNDS) and the National Bureau of Statistics of China by request, respectively, which are presented in the Supplementary Data 1. **d** The control, release and buffer sites are shaded yellow, purple, and blue, respectively, for the urban areas located in Furong District of Changsha. **e** The distribution of adult BG traps and ovitraps at the release, buffer and control sites. BG shows the BG traps. T, B, and C represent the ovitraps located at the release, buffer, and control sites, respectively. **f** Satellite images for the control, release and buffer sites. **g** Total amount of precipitation weekly in Furong district of Changsha from June 21, 2020, to November 22, 2020 is presented as the red line scaled according to the left y-axis. The average number of females per trap per 24 h is presented at the control (blue line) and release sites (green line). The 95% confidence interval of female number at control (blue shading) and release sites (green shading) are scaled according to the right y-axis (*n* = 30 independent trap samples per collection day from June 21, 2020, to November 22, 2020). The total precipitation in the prior week has correlation with average number of females per trap at control site (Pearson correlation: *r* = 0.4871, weekly total precipitation: *n* = 20, control site: *n* = 20, *P* = 0.0294) but has no correlation with the average number of females per trap in release site (Pearson correlation: *r* = 0.4625, weekly total precipitation: *n* = 18, release site: *n* = 18, *P* = 0.0553). **h** The weekly temperature at Changsha from June 21, 2020, to November 22, 2020 is indicated as the orange line with weekly mean minimum and maximum temperatures in Celsius shaded in light orange. The temperature and precipitation data of Furong district in the past 24 h were recorded every day at 16:00 from Weather app (data source: The Weather Channel, https://weather.com).

To explore the independent application of the *Wolbachia*-based IIT without the combination of SIT, a pilot site was established in Changsha city of Hunan Province located in south-central China. This site was selected based on a dramatic increase in the dengue incidence from 2018 (175 cases) to 2019 (746 cases) in Hunan Province (Fig. 1a; Supplementary Fig. 1) according to the local Center for Disease Control (CDC). With approximately no cases recorded from 2010 to 2013, dengue emerged in 2014 and gradually spread to every city of Hunan Province by 2019 (Fig. 1a). Notably, the city with the most dengue cases in 2019 was Changsha, the political and cultural center of Hunan Province (Supplementary Fig. 1a, b) with an area of 11,816 km^2^ and a permanent resident population of 8.3945 million^25^. *Aedes aegypti* was not present, but very high *A. albopictus* densities were noted during mosquito active seasons from June to September, with Breteau indexes greater than the warning level (>20) (Fig. 1b, c). As the highest dengue incidence was traced to Furong District of Changsha in 2019 (Supplementary Fig. 1c, Fig.1d), we selected one of its urban areas located in the Hunan Hybrid Rice Research Center (Fig. 1d–f) as the field site for the trial. Both the release (37,559 m^2^) and control sites (33,333 m^2^) shared similar ecological and natural environments with the subtropical monsoon humid climate (Fig. 1g, h).

### Characterization of prerelease baseline mosquito populations in the field sites

To understand the population dynamics of *A. albopictus* in the field, adults and eggs were monitored weekly with 5 BG-Sentinel traps and 30 ovitraps, respectively, before release and from June 21, 2020 to July 6, 2020 at the release and control sites (Fig. 1e). Consistent with the dynamics noted in 2018 and 2019 in Changsha (Fig. 1b, c), the activity period of the *A. albopictus* population was from June to October (Fig. 1g), and *A. albopictus* became undetectable by the end of November 2020 as the temperature decreased (Fig. 1g, h). Moreover, the weekly number of adult female *A. albopictus* in the control site showed a significant correlation with the weekly total precipitation of the prior week (Pearson correlation: *r* = 0.4871, *n* = 20, *P* = 0.0294) (Fig. 1g), indicating precipitation- and temperature-dependent fluctuations of the local mosquito population.

To validate the appropriateness of the control site for comparison, we confirmed significant correlations of mosquito densities between the release and control sites based on the number of hatched eggs (*r* = 1, *n* = 3, *P* = 0.0028, Supplementary Fig. 2a) and adult females (*r* = 0.9896, *n* = 6, *P* = 0.0002, Supplementary Fig. 2b). Although more mosquitoes were observed at the release site than at the control site, this difference was not statistically significant (larvae: *n* = 3, *P* = 0.7, Supplementary Fig. 2a; adult females: *n* = 6, *P* = 0.5887, Supplementary Fig. 2b). Similarly, there was no significant difference in the egg hatching rates between the release (87.04 ± 1.39%) and control (84.20 ± 1.53%) sites during the pre-release stage (*P* = 0.4000, Supplementary Fig. 3).

### Assessment of HC quality for population suppression in Hunan

**Fig. 2:**
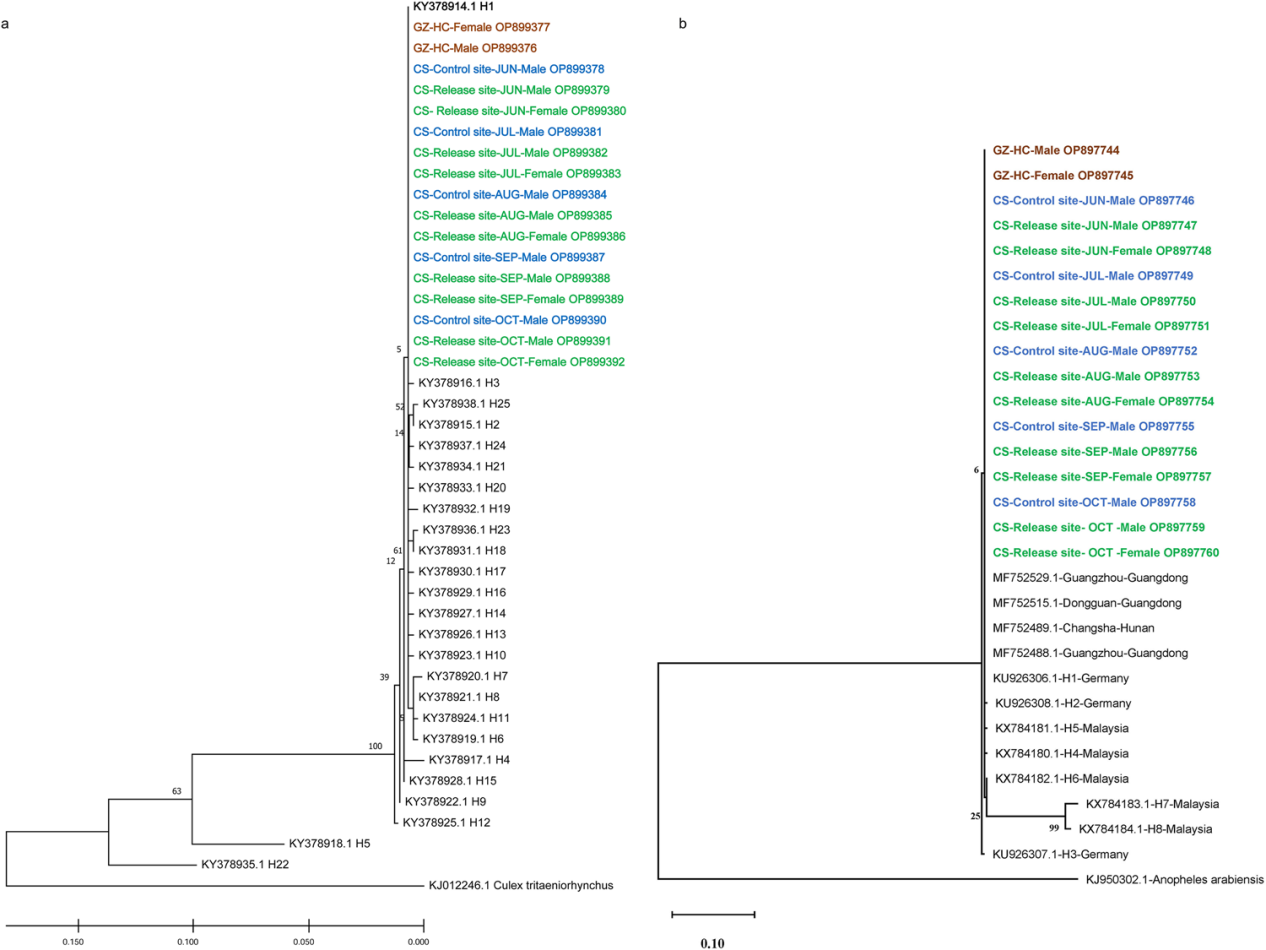
Maximum likelihood phylogenetic analysis of HC and field *A. albopictus*. The maximum likelihood phylogenetic tree of the mitochondrial *COI* gene from *A. albopictus* collected in China (**a**) and the mitochondrial *ND5* gene of *A. albopictus* collected in Hunan and Guangdong Provinces (**b**). The Genbank accession number, gene haplotype (H1-H25 for mitochondrial *COI* gene, H1-H8 for mitochondrial *ND5* gene), and available location of mosquito sample for reference sequences are marked in black color. GZ-HC highlighted in brown color represents the HC line of *A. albopictus* produced in the mass-rearing facility in Guangzhou. The CS-Release site in green color and CS-Control site in blue color indicate the wild *A. albopictus* captured from the release site and control site in Changsha city, respectively, each month from June 2020 to October 2020. The GenBank accession numbers for mitochondrial *ND5* (OP897744-OP897760) and *COI* (OP899376-OP899392) genes are listed after the sample names in the phylogenetic tree.

Despite its previous successful application in population suppression, the *A. albopictus* HC line with infections of *w*Pip, *w*AlbA, and *w*AlbB had a genetic background of mosquitoes from Guangzhou, at a distance of >670 km from the field sites. To investigate whether the population in the pilot sites had isolated genetic diversity that could prevent effective mating with HC males, we performed phylogenetic analysis based upon the genetic distance between wild-type *A. albopictus* collected at the pilot sites and the HC line produced in the Wolbaki mass-rearing facility using alignment of the nucleotide sequence of the mitochondrial cytochrome oxidase I (*COI*) and NADH dehydrogenase subunit 5 (*ND5*) genes. The results showed that both *A. albopictus* from field sites and the HC mosquito belonged to haplotype H1 of the mitochondrial *COI* gene (Fig. 2a). Moreover, *A. albopictus* collected from the release and control sites, representative samples collected from Hunan and Guangdong province^26^ and HC mosquitos were also clustered into haplotype H1 of the mitochondrial *ND5* gene (Fig. 2b). These results suggested that HC line and the wild-type *A. albopictus* at the pilot site had similar genomic background with 100% similarity in mitochondrial *COI* and *ND5* genes (Fig. 2).

**Fig. 3:**
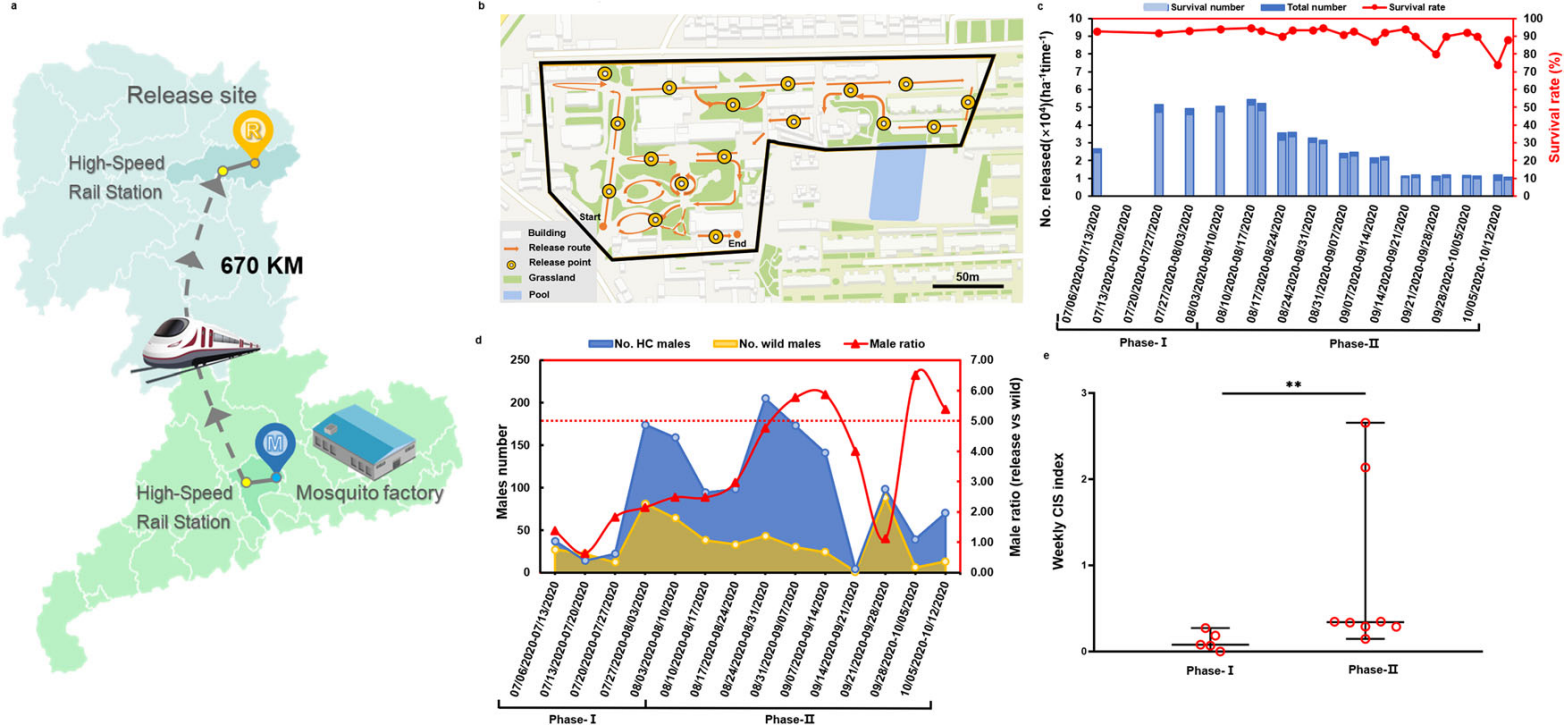
HC mosquito transportation, release route, release number, and mating competitiveness. **a** Transportation of HC mosquitoes from the mosquito factory to the release site. **b** The route for release in the field. The small yellow dots indicate the start and end of release route. The concentric circles present the release spots on the release route. **c** The number of HC males released weekly and the survival rate of HC males at the time of release. The survival rate (red line) is expressed as the proportion of HC males surviving at the release time among the total individuals in the release containers. **d** The ratios of HC males to wild-type males of *A. albopictus* at the release site, calculated using the total number of HC (Release, blue line) and wild male (Wide, yellow line) mosquitos collected from all the BG traps per 24 h in the release site, based on assay of *Wolbachia w*Pip infection via qPCR. The red dashed line represents the target ratio of 5:1 to reach effective suppression based on previous studies^11^. **e** HC male mating competitiveness. The median of weekly CIS indexes in each release phase are shown as the horizontal line with error bar of 95% confidence interval of weekly CIS indexes. Each weekly CIS index value is shown as a red circle. Two-sided Mann–Whitney test, Phase-I: release site: *n* = 5, control site: *n* = 5, *P* = 0.1734; Phase-II: release site: *n* = 8, release site: *n* = 8, *P* = 0.0062**.

During shipping, HC males were placed in a container with an inside temperature of 10 °C based on a previously established protocol^27^. Using ground transportation (high-speed train and vehicle), HC males at 1-day post-emergence were transported from the mosquito factory to the release site within 4–7 h (Fig. 3a) and then released on a fixed route by hand (Fig. 3b). We observed that HC males maintained an average survival rate of 91 ± 1.05%. Once at the outdoor temperature, the mosquitos recovered and flew away within 10 min (Fig. 3c). We further measured the mating competitiveness of HC males in the field based on the weekly capacity to induce the sterility (CIS) index (Fig. 3d, e). Although the median of weekly CIS index was 0.08 in release phase I, it increased to 0.34 in release phase II. These results suggest that HC males had great performance in the field even after long-distance transportation from mass-rearing facilities.

### Strong suppression of the *A. albopictus* population in the field with effects sustained post release

**Fig. 4:**
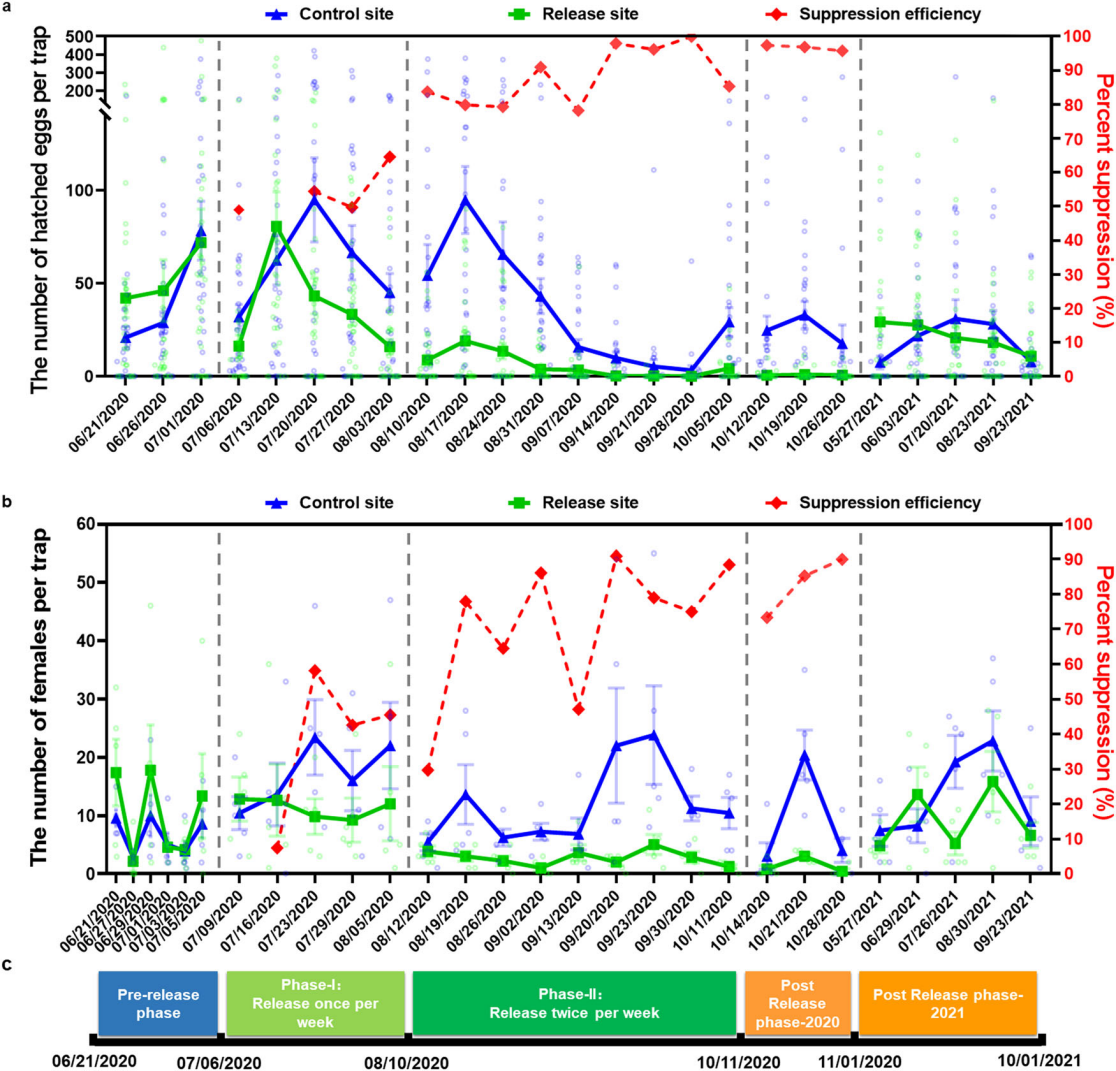
Suppression of egg hatching and female adults by HC release. **a** The number of hatched eggs of *A. albopictus* per trap and suppression efficiency at the release site compared to the control site. The blue and green solid lines indicate the average number of hatched eggs (trap^−1^ week^−1^) in the control and release sites, respectively. The red dashed line presents the suppression efficiency of the release site compared to the control site. Each light blue and light green circle corresponding to a number of hatched eggs from an individual retrieved ovitrap in control site and release site, respectively. Two-sided Mann–Whitney test, pre-release phase: Release site: *n* = 90, Control site: *n* = 90, *P* = 0.9269; phase I: Release site: *n* = 143, Control site: *n* = 148, *P* = 0.0119; phase II: Release site: *n* = 269, Control site: *n* = 262, *P* = 5.5047 × 10^−14^; post-release phase-2020: Release site: *n* = 90, Control site: *n* = 89, *P* = 2.7849 × 10^−10^; post-release phase-2021: Release site: *n* = 147, Control site: *n* = 150, *P* = 0.1524. The vertical dotted lines indicate the interval of each release phase. **b** The number of females per trap per 24 h at the release site (light green circle) and control site (light blue circle). The green and blue solid line represent the average number of females per trap per 24 h in the release and control sites, respectively. The red dotted line indicates the suppression efficiency at release sites relative to control sites. Two-sided Mann–Whitney test, pre-release phase: Release site: *n* = 30, Control site: *n* = 30, *P* = 0.9441; phase I: Release site: *n* = 25, Control site: *n* = 25, *P* = 0.0391; phase II: Release site: *n* = 45, Control site: *n* = 45, *P* = 7.1467 × 10^−10^; post-release phase-2020: Release site: *n* = 15, Control site: *n* = 15, *P* = 0.0294; post-release phase-2021: release site: *n* = 25, control site: *n* = 25, *P* = 0.161. **c** Release schedule. The date on x-axis is the starting date of monitoring via ovitraps (a) and BG traps (**b**). The error bars present the standard error of mean.

**Fig. 5:**
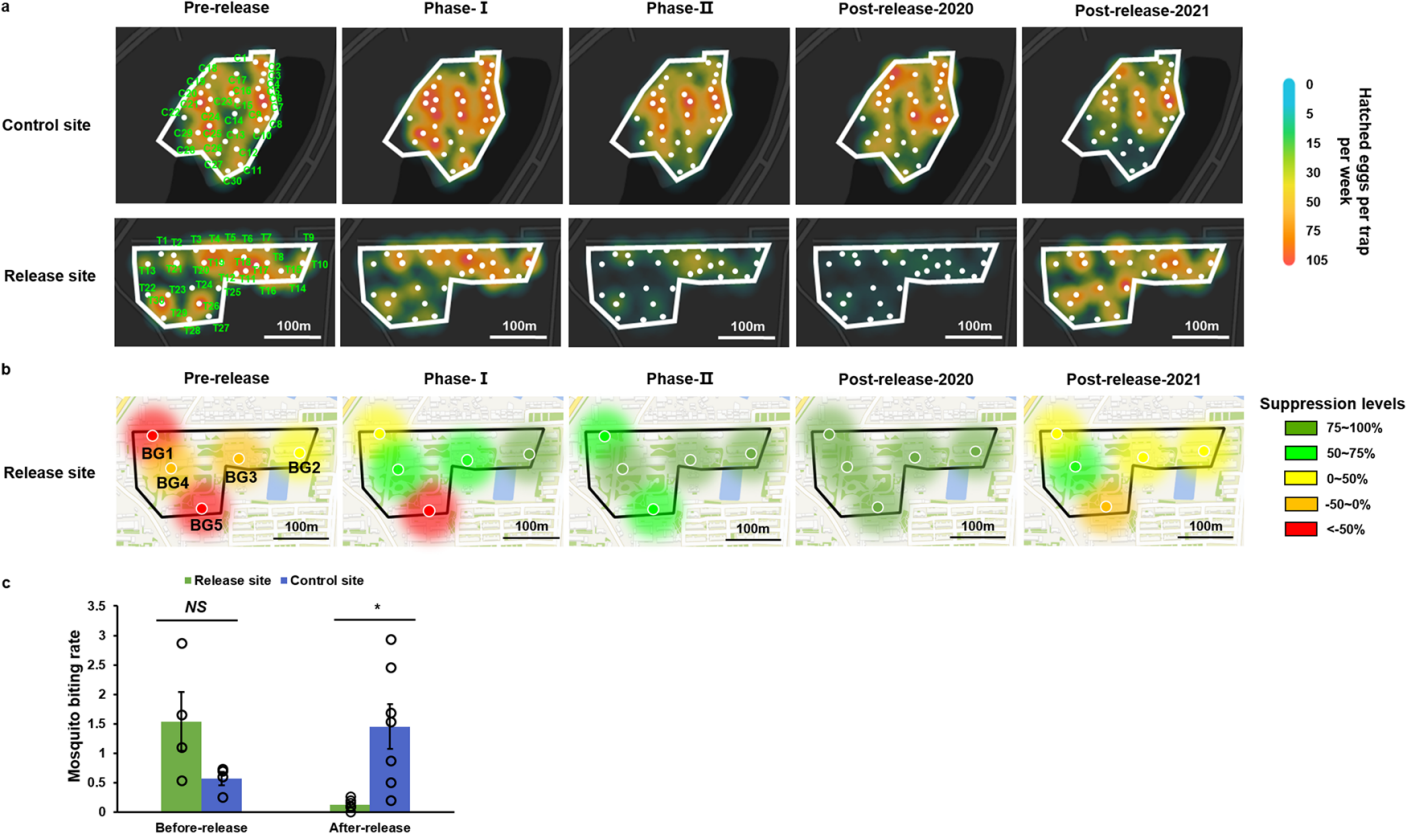
Spatial dynamics of larval and adult population suppression. **a** Spatial-temporal dynamics of weekly hatched eggs per trap at the release site and control site for each release phase. The white dots indicate the location of the ovitrap at the release site and control site. The different color indicates the average number of weekly hatched eggs for each trap during each phase. **b** Spatial-temporal dynamics of suppression on *A. albopictus* female adults at the release site for each release phase. **c** Reduction in mosquito biting. Mosquito human landing catches in the release and control sites. Data are shown as the means ± SEMs. Before-release: June 21, 2020–July 6, 2020; After-release: July 6, 2020–November 1, 2020. Two-sided paired t test, Before-release, *t* = 1.778, release site: *n* = 4, control site: *n* = 4, 3 degrees of freedom, *P* = 0.1734; After-release, *t* = 3.565, release site: *n* = 7, control site: *n* = 7, 6 degrees of freedom, *P* = 0.0119*.

We started with one release each week of 2.64 × 10^4^ HC males per hectare in phase I from July 6, 2020 to August 10, 2020 (Fig. 3c). In the first week after release, immediate suppression to 49.04% was observed based on the average number of hatched eggs per trap (Fig. 4a), but no suppression was observed if measured by the number of female adults/traps/24 h (Fig. 4b). No release was performed in the second week. Correspondingly, the suppression effect on egg hatching observed in Week 1 disappeared in Week 2 (Fig. 4a, c). The release was resumed from the third week to the fifth week (July 20, 2020 to August 10, 2020), with approximately 5 × 10^4^ males per hectare released weekly (Fig. 3c). Although the ratio of released HC males to wild-type males remained at 2:1 for three consecutive weeks (July 20, 2020 to August 10, 2020, Fig. 3d), the suppression efficacy increased steadily and reached 65% and 46% in Week 5 (August 3, 2020 to August 10, 2020) based on the numbers of hatched eggs and trapped female adults, respectively (Fig. 4). Variation in the suppression level was noted across different monitoring sites, with stronger suppression of egg hatching in the southwest than in the northeast (Fig. 5a, b). However, BG trap locations 1 and 5 showed a slower suppression of female adults even with a strong suppression of hatched eggs (Fig. 5a, b). Overall, mild suppression of *A. albopictus* was noted in phase I, and the population was reduced by 37% and 34% based on the numbers of hatched eggs (*P* = 0.0119, Fig. 4a) and adult females (*P* = 0.0391, Fig. 4b), respectively.

To boost the suppression efficacy, the release frequency was increased to twice per week in phase II from August 10, 2020 to October 12, 2020 (Fig. 3c). The number of released HC males was increased to 10.63 × 10^4^ males per hectare in Week 6 (August 10, 2020 to August 17, 2020) and then gradually declined from 7.11 × 10^4^ to 4.37 × 10^4^ males per hectare from Week 7 to 10 (August 17, 2020 to September 14, 2020, Fig. 3c), resulting in a ratio of HC males to wild-type males of 5.77:1 in Week 9 (August 31, 2020 to September 7, 2020, Fig. 3d). Consequently, the suppression efficacy reached 91% and 86% based on the numbers of hatched eggs and adult females in Week 9, respectively (Fig. 4). From Week 11 to 14 (September 14, 2020 to October 12, 2020), the released HC males decreased to approximately 2.3 × 10^4^ males per hectare weekly (Fig. 3c), and the strong suppression was maintained in the final week of phase II, with efficacies of 85% and 88% for hatched eggs and adult females, respectively (Fig. 4). During phase II, average reductions of 83% (*P* = 5.5047 × 10^−14^, Fig. 4a) and 77% (*P* = 7.1467 × 10^−10^, Fig. 4b) were noted in the larval and female populations, respectively, at the release site. A greater than 75% suppression level was consistently observed at BG trap monitoring points 2 and 4, whereas the average suppression level reached 65% and 57% at BG trap monitoring points 1 and 5, respectively (Fig. 5b). Interestingly, the areas with a lower number of hatched eggs at the release site were located around the release route in phase II (Fig. 5a), whereas the areas with a lower number of hatched eggs among the ovitrap monitoring points at the control site were those with fewer human activities.

We continued monitoring the populations post release until the end of the mosquito season, from October 12, 2020 to November 1, 2020 (Fig. 4c), to investigate the duration of suppression. In total, 97% suppression of hatched eggs (*P* = 2.7849 × 10^−10^, Fig. 4a) and 85% suppression of adult females (*P* = 0.029, Fig. 4b) were noted for the three consecutive weeks at the release site. An analysis of spatial population dynamics showed that strong suppression occurred at each ovitrap and BG trap monitoring site across the entire release area (Fig. 5a, b). However, the suppression effect was not observed at the release site in 2021 based on the monitoring of females and hatched eggs (Figs. 4, 5).

To estimate the epidemiological importance of population suppression on dengue transmission, we used a human landing assay to measure and compare the mosquito biting rates between the release and control sites during and after release. Before release, no significant difference was noted between the two sites, with a trend of a higher biting rate at the release site relative to the control site. However, after release, mosquito biting was reduced by 94% at the release site compared to the control site (*P* = 0.0119, Fig. 5c, Supplementary Fig. 4). These results indicate that IIT has the potential to prevent dengue virus transmission by reducing the mosquito biting rate.

### Prevention of population replacement with successful suppression by standalone IIT

**Fig. 6:**
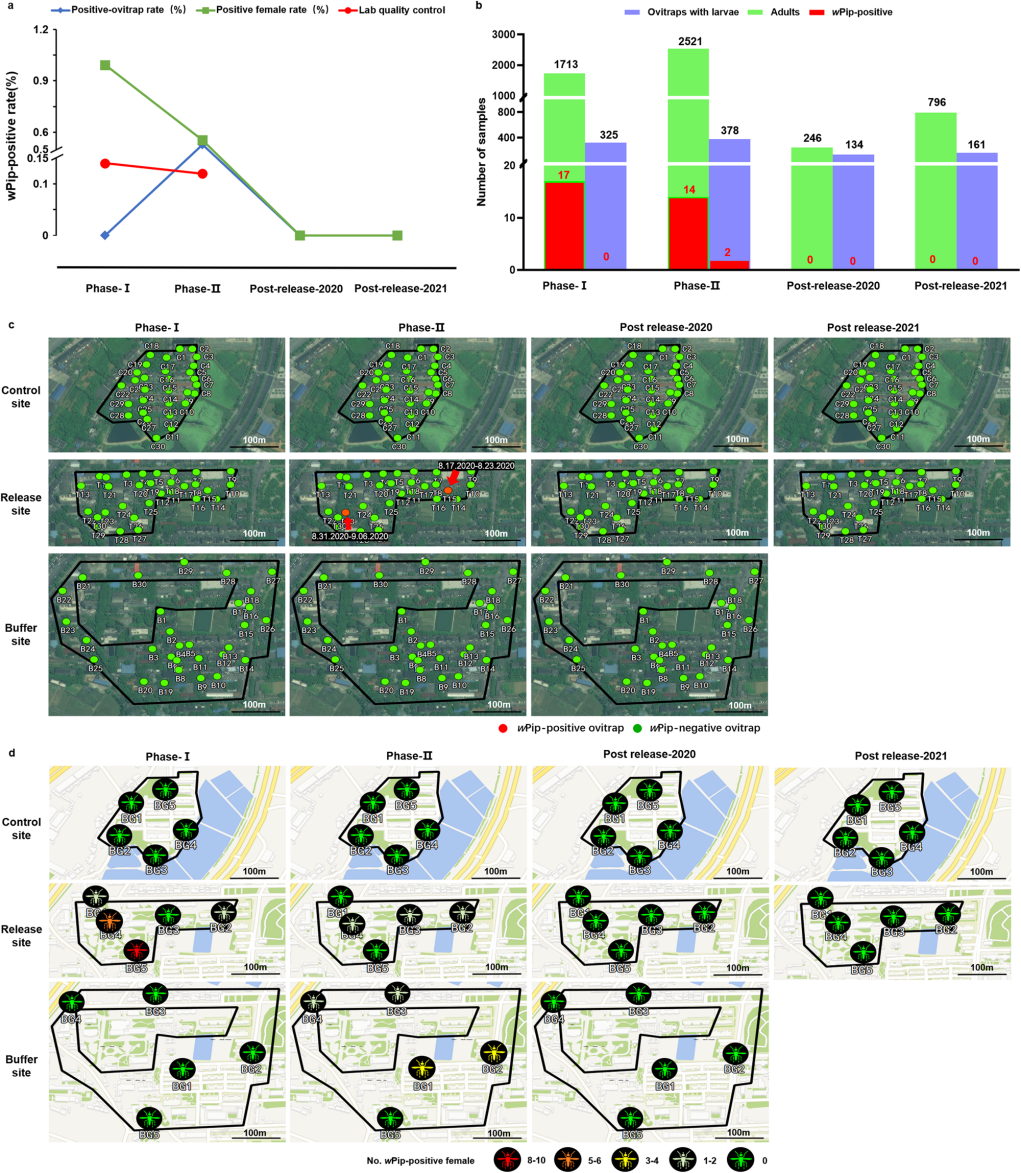
The presence and spatial-temporal dynamics of *w*Pip-positive samples in the field. **a** Comparison of the *w*Pip-positive female rates detected in HC males in the mass-rearing facility (laboratory quality control) and the positive female rate monitored in the adults collected via BG traps as well as the *w*Pip-positive rate of ovitraps from the field sites. **b** The *w*Pip-positive females and larvae detected in the field. Ovitraps with larvae: the number of retrieved ovitraps containing eggs that were hatched to larvae in the laboratory; Adults: the number of adult *A. albopictus* collected via BG traps; *w*Pip-positive: the number of *w*Pip-positive female adult or pooled larva samples. **c** Spatial distribution of *w*Pip-positive larvae collected from the control site, release site and buffer site, shown as red dots and marked with the collection time in the format of “Month-Day-Year”. **d** Spatial distribution of *w*Pip-positive adult females collected from the release site, control site and buffer site, shown as mosquito symbols and colored according to the number of *w*Pip-positive females. No significant difference in the weekly number of *w*Pip-positive females (two-sided Mann–Whitney test, phase-I: *n* = 5, phase-II: *n* = 9, *P* = 0.1069) and larvae (two-sided Mann–Whitney test, phase-I: *n* = 5, phase-II: *n* = 9, *P* = 0.5055) was observed in phase II compared to phase I at the release site and buffer site, and there was no evidence that the number of *w*Pip-positive female and larvae mosquitoes increased over time. Phase I: July 6, 2020–August 10, 2020; Phase II: August 10, 2020–October 12, 2020; Post-release-2020: October 12, 2020–November 1, 2020; Post-release-2021: May 2021 to September 2021.

Combining IIT with SIT was previously used to prevent the risk of population replacement due to imperfect sex separation in mass production. Given numerous environmental factors restricting the establishment of *Wolbachia*, we hypothesized that standalone IIT can effectively suppress the field population without causing population replacement even under our current sex separation precision. The presence of residual females was examined as a quality control during the mass production of HC males to assess the accidental release of HC females into the field. We observed a mean of 0.13 ± 0.01% (standard error of the mean (SEM)) female contamination rate in the HC males during this trial (Fig. 6a). To monitor the risk of establishment of HC mosquitoes in the field, the caught females and the larvae developed from the eggs collected in the ovitraps from the release site, its surrounding buffer area and the control site were sacrificed to determine *Wolbachia w*Pip infection. During phase I, all the tested larvae obtained from hatching eggs collected in 325 ovitraps were negative, but 17 out of 1,713 females (0.99%) collected from the release site were positive (Fig. 6b). In phase II, two ovitraps with *w*Pip-positive larvae samples were detected among 378 ovitraps (0.53%) with newly hatched larvae, and 14 positive females were detected among 2521 individuals (0.56%) collected from release and buffer areas (Fig. 6b). No positive larvae or females were further detected during the post-release phase in either 2020 or 2021 (Fig. 6c, d), indicating no establishment of HC mosquitoes in the field.

To evaluate the potential impacts of population suppression on the genetic diversity of *A. albopictus* at the pilot sites, wild-type *A. albopictus* were collected via BG traps at both the control site and release sites each month during release, and their mitochondrial *COI* and *ND5* genes were sequenced. Phylogenetic analysis showed that all tested mosquitoes clustered into the clade representing haplotype H1 of mitochondrial *COI* and *ND5* genes (Fig. 2). Haplotype H1 from either mitochondrial *COI* or *ND5* gene is a widely distributed dominant haplotype in mainland China^26,28^, supporting that no impact of IIT implementation on the genetic diversity of *A. albopictus* at the pilot sites.

## Discussion

Combining IIT with SIT by release of HC males previously resulted in strong suppression of *A. albopictus* populations in two Guangzhou islands. In this study, we conducted the application of a standalone IIT for mosquito population suppression in urban areas with the risk of population replacement prevented. The release of HC males resulted in reducing the number of hatched eggs by 97% and the female adult density by 85%, even during the post-release period in 2020. In addition, the mosquito biting rate was reduced by 94% at the release site compared to the control site. The strong suppression was maintained even after release stopped until the end of the mosquito season. None of the *w*Pip-positive individuals were detected after the release period, although residual females present in the released males had not been sterilized by irradiation, *w*Pip-positive female contamination ranging from 0.56% to 0.99% was noted, and two positive larval pools were detected in the field. Our results also showed the ability of HC males to maintain high survivorship and mating competitiveness after long-distance transportation at 10 °C. The HC line shared the same genomic background with the wild-type *A. albopictus* in the field, although they were derived from two geographic locations that were 670 km apart.

Although spread of the *Wolbachia* triple-strain infection carried by HC mosquitos into the field population may result in permanent reduction of dengue transmission owing to its capacity to reduce vector competence^11^, population suppression may fail if HC males and a few residual females are released continuously as mating between individuals with the same *Wolbachia* strains is compatible. To sustain population suppression or accomplish population elimination, two current strategies for preventing this risk include the use of either irradiation to sterilize residual females or artificial intelligence to increase the accuracy of sex separation. However, both methods will increase the cost of the suppression program due to the requirement for expensive equipment, including an irradiator custom-made for treating mosquito and a sex sorter integrated with a machine learning function. Furthermore, evidence has indicated a potential irradiation-related reduction in male quality, which is particularly sensitive to the dose of irradiation^11,12^, and even the above advanced sex sorter cannot completely prevent population replacement when the suppression effect is maximized to near elimination^13^. The upgraded automatic mosquito pupae sex sorter used in our mass-rearing facility enabled us to scale up the production with an approximate 80% male recovery rate and an average 0.13% female contamination rate. Theoretically, there was a chance for replacement to occur, as an estimated 2705 fertile HC females were released into the field based on a total of 2.08 × 10^6^ HC males released during the trial. However, with the exception of a very few positive females and larvae detected in the field during the release, all collected samples were negative post-release in 2020–2021, indicating these released females were eliminated from the population. Based on the number of release males, the observed ratio of HC to wild-type males, and assuming equal sex ratio in the field, we estimated the target field population would be as large as 1 million during phase II at the release site. The total number of males and females of target field population was estimated as follows: the release number of HC males is multiplied by the observed ratio of wild-type males to HC males, and then multiplied by two. Although very strong suppression of *A. albopictus* was accomplished, the number of the remaining wild-type female mosquitoes in the field was much higher than that of accidentally released females. Consequently, the female infection frequency, approximately 1.25% based on 5 HC females among 399 females collected at the release site in phase II, is still far below the required invasion threshold. Our results indicate that these released HC females may not successfully breed under field environmental conditions such that the triple infection was unable to pass the invasion threshold and was eventually removed from the population. Elimination of *Wolbachia* was previously observed in *A. aegypti* laboratory populations when the *Wolbachia* infection frequency was less than 20%^29^. Sustainable establishment of *w*MelPop failed at field sites in Vietnam and Australia due to a substantial reduction in mosquito lifespan associated with *w*MelPop^20^. Although *w*Mel had been successfully established in the field for more than 10 years in Australia, it remained in less than 5.1% of mosquitoes four years after their release in tropical central Vietnam, with the most rapid declines in *Wolbachia* infection prevalence correlated with the onset of the hot dry season owing to the susceptibility of the *w*Mel strain to heat stress^21^. Negative effects of *Wolbachia* on the longevity and viability of eggs produced by transinfected *Aedes* mosquitoes have been reported^18^. *Wolbachia* density was reported to decrease across egg storage time, and females that developed from quiescence eggs showed fertility loss^19^. Low temperatures also suppressed *Wolbachia* density in transinfected *A. aegypti*^22^. Considering that the average daily minimum temperature is less than 10 °C from November to March at the release site, it is likely that most of the eggs laid by accidentally released HC females either did not survive to the next mosquito season or failed to develop into *w*Pip-infected fertile females. Due to these fitness costs, *Wolbachia* will be eradicated from the population when the infection frequency is below the invasion threshold, as supported by both mathematical models and empirical experimental evidence^29–31^. Our results suggest that IIT alone can be used for population suppression without causing population replacement, particularly in subtropical regions, such as Furong district of Changsha city, Hunan Province with a mean annual temperature of approximately 16 °C and a long winter (approximately 5 months with an average daily minimum temperature of less than 10 °C) between mosquito seasons, or tropical regions with an extended dry season during which egg quiescence is essential for mosquito survival. In Singapore’s trial, however, the steady detection of *Wolbachia*-positive females in the field *A. aegypti* population was reported when the targeted population was close to elimination^13^. This result is consistent with the predication that, when population suppression is high and approaches the elimination of the population, any accidentally released fertile females will inevitably constitute the majority of the field population and any invasion threshold is irrelevant and will be exceeded^12^. Thus, if the goal is to eliminate a population or when the near-elimination of the wild population is attained, particularly in tropical areas with dengue transmission year around, a combination of IIT and SIT or a hybrid of IIT and SIT remains an option that will warrant success^12,13,32^ for the prevention or mitigation of population replacement.

The success of reducing the public health burden of dengue requires a vector control tool that can be applied in urban areas. Following strong suppression of the *A. albopictus* population on two islands, we conducted this field trial to test the efficacy of IIT on population suppression in urban areas to gather empirical data for guiding the next operation of IIT for dengue control. For urban areas with complex topography and interactions between social and natural factors, the precise management of the release route and the release number and frequency is essential during the deployment of IIT. We found that the high suppression effect along with the release route might be diluted with the increasing distance from the release route. While HC males caused strong suppression at the release site, some HC males flew outside, contributing to a weaker and unstable suppression effect observed in buffer area. Moreover, the suppression effect could be affected by the type of mosquito habitats and immigration of mosquitoes from neighboring areas. We observed that the BG trap locations 1 and 5 had slower suppression effect than the BG trap locations 2 and 3, although all the four BG traps located near the release points. This difference may be caused by specific mosquito habitats next to locations 1 and 5. For example, location 1 was close to the shrubs surrounding a garbage transfer station while location 5 had a bamboo grove nearby buildings, resulting in more mosquito immigrated to locations 1 and 5 than locations 2 and 3. It is worth noting that the distance between each location was over 100 m, within an estimated flight range of *Aedes* mosquitoes^33^ (BG trap 1 to 2: 270 m, BG trap 5 to 2: 210 m, BG trap 1 to 3: 160 m, and BG trap 5 to 3: 120 m). Based on a background investigation of field mosquito density, the natural environment and human activity, we designed a release route to maximally cover mosquito habitats and sites with intensive human activity and a high density of mosquitoes. Given that the public would prefer to have the lowest number of released mosquitoes in urban areas even if the HC males do not bite, we examined whether a release dose lower than the previously used dose was sufficient to suppress the population. Although the number of released HC males gradually decreased during release phase II compared to release phase I, the increase in release frequency to twice per week resulted in enhanced suppression efficiency with an effect persistent through the post-release phase. These results indicate that highly effective suppression can be accomplished through the release of a lower number of HC males twice per week rather than of a higher number once a week. Importantly, the average number of HC males released/Ha/week in this field trial was only 28% of that reported in a previous field trial in Guangzhou although the release sites had similar mosquito densities before release and similar suppression effects were reached at the end of trials^11^. Consistently, we observed high mating competitiveness in the field in phase II of this trial even though mosquitoes experienced long-distance transportation under low temperature. It remains to be determined whether this increase in efficacy is due to the removal of the radiation step in mass production. We observed that the release of HC males caused a rapid decrease in egg hatching with instant suppression detected in the first week of phase I. A release frequency of once-per week with an HC to wild-type male ratio of 2:1 resulted in a 65% reduction in egg hatching at the end of phase I. This result suggests that the average number of hatched larvae could be used as a sensitive and fast-response index for IIT.

Deployment of IIT would require long-distance transportation of HC males from our mass-rearing facility to other cities in China at risk of dengue. We previously found that the mating competitiveness of HC males would not be affected after maintaining the mosquitoes at 10 °C for 3 h, and only minor negative impacts were noted after storage at 10 °C for 24 h^27^. Consistent with these results, we observed a high survival rate and strong mating competitiveness of HC males after 4 to 7 h of transportation from Guangzhou to Changsha under low temperature conditions. Of note, although large variation among the weekly CIS was observed in phase II probably due to the climate change from summer to early fall, a great field competitiveness of HC males was seen with the increased release frequency, based on the increase in the weekly mean CIS index by 6.8-fold in phase II relative to phase I. As wild males emerged every day while HC males declined due to daily mortality^34^, two releases per week are expected to mitigate this age-related disadvantage of released males in the field and thus increase the cost-effectiveness of the IIT program.

It is unknown how the observed population suppression will impact dengue transmission. We observed a 94% decrease in mosquito biting at the release site compared to the control site, supporting its potential effect in dengue control. Indeed, there was no dengue incidence at the release site in 2020. However, dengue cases also declined in both Hunan Province and the mainland China, likely due to the decrease in international travel and outdoor activities caused by the global COVID-19 pandemic. Further study will determine whether a standalone IIT can be scaled up to reduce dengue transmission in Furong district of Changsha city.

Overall, our results show that standalone IIT can effectively suppress mosquito populations in urban areas with subtropical monsoon climates and that the risk of population replacement can be prevented even without integration with other technologies to remove residual females in the released males as long as the goal is not population elimination. Strong suppression with a lower dose compared with our previous trials of HC male releases highlights the efficacy of IIT in area-wide implementation for dengue control. The feasibility of long-distance transportation of HC males under low temperature conditions further aids the deployment of IIT across a large area at risk of dengue with one regional mosquito mass-rearing facility. Our future work includes using IIT to remove dengue hot spots in urban areas, measuring its direct impact on dengue transmission, and exploring an effective model to integrate population suppression with population replacement to maximize the power of *Wolbachia*-based vector control.

## Methods

### Description of the study areas

The control (33,333 m^2^), release (37,559 m^2^), and buffer (107,199 m^2^) sites were established in the Fengshou Garden Community (28°12′19.33″N, 113°4**′**34.65″E), Hunan Hybrid Rice Research Center (28°12′6.23″N, 113°4′39.97″E), and Fengyu Community (28°12′1.94″N, 113°4′47.10″E), respectively, in Changsha, Hunan Province (Fig. 1d–f). There was an eight-lane highway and a small lake with a 230-m span between the release and control sites. The buffer site was set up to surround the release site to monitor the spillover effect of HC male release on the neighboring area. Under a subtropical monsoon climate with a mean annual precipitation of 1300–1500 mm and a mean annual temperature of 16.3 °C (https://weather.com), all the field sites shared a common ecological environment, including office buildings (7 in the release site, 7 in the buffer site, and ~1 in the control site), restaurants (1 in the release site, 2 in the buffer site, and 1 in the control site), sports grounds (1 per each site), residential buildings (3 in the release site, 11 in the control site, and 28 in the buffer site), and green belts. The field trials were authorized by the Ministry of Agriculture of the People’s Republic of China (SY2019092).

### HC male mass-rearing and low temperature transportation

The HC line of *A. albopictus* carried a triple-*Wolbachia* strain infection of *w*Pip, *w*AlbA, and *w*AlbB^11^. Adult female oviposition, egg hatching, larval rearing, sex separation, quality control, and packaging were performed at the mass-rearing facility of Guangzhou Wolbaki Biotech Co., Ltd. (Wolbaki) according to a standard protocol^11,35^. Briefly, the 5 to 6 days old adult females were fed with sheep blood (provided by a local abattoir) containing ATP (5 g/L). At day 2 post blood meal, the oviposition site was provided for the females in the mosquito cage, which was a plastic cup filled with one third of a cup of water and attached a piece of oviposition paper to the inside wall of cup. The egg collection was performed for two days to allow the females to lay eggs on the wet oviposition paper. Followed by removing the water from the oviposition cup, the eggs were matured under the standard condition at 80 ± 5% humidity and 28 ± 1°C for a week. Subsequently, the oviposition paper with eggs was placed in the container filled with water for egg hatching. The newly hatched first instar larvae were transferred to the tray (58 × 38 × 4 cm) filled with ~4 L water (1.5 cm depth) to achieve a density of 6600 larvae per tray and fed daily with nutrient solution containing 60% beef liver powder, 30% shrimp powder and 10% yeast for continuous 6 days. From day 7 post hatching, the larvae were gradually developed to pupa in the water without nutrient solution. At day 8 post hatching, the identification and separation of HC male pupae were conducted by an automatic mosquito pupae sex sorter developed by Wolbaki with improved efficiency (separation of 75,000 male pupae per hour) and accuracy^23^ (female contamination rate less than 0.5%). Subsequently, the male pupae were transferred to mosquito cages (30 × 30 × 30 cm) with routinely provided 10% sugar solution. At day 1 post emergence, adult HC males were subjected to lab quality control by random inspection to obtain the female contamination rate based on morphological characteristics^11^. In short, we randomly sampled anaesthetized adult mosquitoes thrice. Each time, the sex of 1000 mosquitoes was manually determined via microscopic examination of their terminalia. Then, HC males were packaged into a plastic container (20 × 14 × 3.5 cm) with clear plastic vented lids at a density of 8000 mosquitoes per container. Immediately after packing, mosquitoes were delivered by a high-speed train and vehicle from the mosquito factory to the release site over a distance of approximately 670 km and a duration of 4–7 h (Fig. 3a). During shipping, mosquitoes were maintained at 10 ± 1 °C in a portable cooler (Kbcool, Zhejiang, China) with a temperature indicator^27^.

### Male HC release

HC male release was performed every Monday during phase I and every Monday and Friday during phase II. On the scheduled day, HC mosquitos were shipped out in the early morning and arrived at approximately 15:00 pm to 18:00 pm on the same day. Upon arrival at the release site, containers were removed from the portable cooler to enable mosquito to recover at the outdoor temperature. During release, containers were opened by removing the vented lids and mosquitoes were allowed to fly away by themselves. Release was performed by staff walking along the fixed route including 17 release spots, 35 m apart (Fig. 3b), and the process took approximately 0.5 h. At the end of the release, the numbers of mosquitoes that could not fly were recorded. Mosquito survival rates (Sr%) were calculated based on the following equation, where Nr and Nn are the numbers of all HC males for release and non-flyable HC males, respectively.

1 $${Sr} \% \,=\,({Nr}\,-\,{Nn})/{Nr}\,\times\, 100 \%$$

The number of mosquitoes for release and the release frequency were empirically adjusted according to the ratios of HC males to wild males monitored at the release site from the previous week. The release included two phases: phase I (July 6, 2020 to August 10, 2020) with release once a week and phase II (August 10, 2020 to October 12, 2020) with release twice a week, with one exception of no release in the second week (July 13, 2020 to July 20, 2020).

### Monitoring of *A. albopictus* population suppression at the adult stage

*A. albopictus* densities were monitored at the control and release sites from June 21, 2020 to November 22, 2020. Continuous 24-h adult monitoring was performed from 48 to 96 h post-release in each week using 5 BG-Sentinel traps (Biogents) placed at the release and control sites approximately 100 m apart. In addition, 5 BG-Sentinel traps were placed at the buffer site approximately 170 meters apart.

Morphological identification and sex determination of collected adults were performed^11^. Subsequently, HC mosquito was distinguished from wild-type *A. albopictus* in adult samples by detection of *Wolbachia w*Pip infection using PCR. A unique code on each BG-Sentinel trap was used to track the location of the sample collected. The number of females per trap per 24 h was recorded for each retrieved BG trap. These values were used to calculated the average number of females per trap per 24 h, which is defined by dividing the total number of females collected in 24 h by the number of retrieved BG traps, and were compared weekly between the release and control sites. The suppression efficiency of the IIT application on *A. albopictus* adult (SEa) was calculated based on the following equation, where Fc and Fr are the average numbers of females per trap per 24 h at the control and release sites, respectively.

2 $$\,{SEa}\,=\,\frac{{Fc}\,-\,{Fr}}{{Fc}}\,\times\, 100 \%$$

The suppression level at each BG site was presented on a map to visually display the spatiotemporal dynamics of *A. albopictus* adult population using Bigemap v30.0.0.0 software (www.bigemap.com) with the thermal radius set at 50 m. Maps are derived from Map World (https://www.tianditu.gov.cn).

### Monitoring of *A. albopictus* population suppression at the immature stage

*A. albopictus* populations at the immature stage, including eggs and larvae, were monitored based on weekly egg collection from 30 ovitraps (Ningbo Bangning Vector Biological Control Products Co., Ltd.) placed at the control and release sites approximately 40 meters apart, with additional 30 ovitraps in the buffer area 50 meters apart. Each ovitrap contained approximately 50 mL of bamboo leaf-soaked water to attract females to oviposit on a piece of filter paper (10 × 6 cm) placed around its internal wall. These ovitraps were deployed every Monday and then retrieved and replaced one week later. The eggs from the retrieved ovitraps were hatched in the incubator (Kuntian, 303-3AB) at 28 ± 1 °C for 7 days. The numbers of hatched eggs and total eggs in each retrieved ovitrap were recorded under a dissecting microscope. Then, the hatched larvae from the same individual ovitrap were pooled, with 1–50 larvae per pool, and used for assay of *w*Pip infection and validation of *A. albopictus* species via PCR.

To measure the suppression efficiency of the IIT application on *A. albopictus* population at the immature stage, the average numbers of hatched eggs per trap, calculated by dividing the total number of hatched eggs by the number of retrieved ovitraps, were compared weekly between the release and control sites. The suppression efficiency at the immature stage (SEi) was calculated based on the following equation, where Hc and Hr are the average numbers of hatched eggs per trap at the control and release sites, respectively.

3 $$\,{SEi}\,=\,\frac{{Hc}\,-\,{Hr}}{{Hc}}\,\times\, 100 \%$$

To visualize the dynamic of *A. albopictus* eggs, a heatmap was drawn for each release stage based on the number and location of hatched eggs at the release and control sites using Map Lab (https://maplab.amap.com) with the thermal radius set at 20 meters. Maps are derived from Map World (https://www.tianditu.gov.cn).

### Genomic DNA extraction

Samples of adult mosquitoes and larvae were homogenized in 50 µL STE buffer (Solarbio T1110) on ice for 2 min with a disposable sterile enzyme-free pestle (JET, csp001002). Subsequently, 5 μL of 20 mg/mL proteinase K (Coolaber, CP9191) was added to each sample followed by incubation at 55 °C for 3 h and then at 95 °C for 10 min. The processed samples were used for subsequent PCR assays.

### Detection of *Wolbachia w*Pip infection using quantitative PCR

The sexes of *A. albopictus* adults were determined based on the morphological characterization^11^. A total of 5276 individual adults and 896 pooled larvae were assayed for *Wolbachia w*Pip infection using qPCR, performed on a CFX96 real-time PCR detection system (Bio-Rad) using primers specific for the *orf7* gene of *Wolbachia w*Pip^11^ with wild-type *A. albopictus* and HC mosquitoes as the negative and positive controls, respectively. Based on the qPCR results, we recorded the number of HC males and females, indicated by positive amplification of *w*Pip *orf7*, as well as the number of wild males and females, indicated by negative amplification of *w*Pip *orf7*. To identify any potential contamination of *A. albopictus* larval samples by *Culex*, larval gDNA with positive amplification of *w*Pip *orf7* was used in the subsequent qPCR assay with the primers for the *ribosomal protein S6* (*RPS6*) gene of *Culex* mosquitoes^11^.The qPCR was performed using a TB Green Premix Ex Taq (Tli RNase H Plus) Kit (Takara, RR820A, China), with a program consisted of 1 min at 95 °C, followed by 40 cycles of 10 s at 95 °C, 30 s at 55 °C, and 30 s at 72 °C. Finally, the melting curve was generated from 65 °C to 95 °C. HC larvae were identified when the samples had positive amplification of *w*Pip *orf7* and negative amplification of *Culex RPS6*.

### Assay of HC male mating competitiveness in the field

The relative mating competitiveness between the released HC males and wild *A. albopictus* males was evaluated weekly based on the capacity to induce sterility (CIS) index. The CIS index was calculated using the following equation^34^:

4 $${{{\rm{CIS}}}}\; {{{\rm{Index}}}} = {{{\rm{W/H}}}}\times[({{{\rm{PC}}}}- {{{\rm{PR}}}})/{{{\rm{PR}}}}],$$

where W and H are the numbers of wild males and HC males collected via BG traps at the release site weekly, respectively, and PC and PR are the average percentage of egg hatching at the control site and release site weekly, respectively. Based on the detection of *Wolbachia w*Pip infection via qPCR, the numbers of wild and HC males were recorded weekly as described above. The average percentage of egg hatching per ovitrap was measured by dividing the total number of hatched eggs by the total number of collected eggs for each site weekly. Since the denominator of the above equations cannot be zero, the data at the thirteenth week of release were excluded due to none of egg hatching in the release site (PR = 0).

### Monitoring of the risk of population replacement

The risk of population replacement caused by a small number of HC females mixed within the released HC males was monitored weekly from July 6, 2020 to November 22, 2020 and from May 27, 2021 to October 1, 2021 based on the detection of *w*Pip by PCR in the mosquito adults and larvae collected from the release site. The two indicators were used to monitor the risk: (i) the proportion of ovitraps with *w*Pip-positive larvae in the total ovitraps with larvae detected and (ii) the proportion of *w*Pip-positive female adult in the total collected females. The dynamics of *w*Pip-positive larvae and *w*Pip-positive female adults was further tracked according to their trap locations in each stage during 2020–2021.

### Mosquito human landing assay

Mosquito biting ratios, defined as the average number of female *A. albopictus* caught per person per minute, were compared between the release and control sites from June 27, 2020, to October 22, 2020, according to the protocol approved by the Ethics Committee on Biomedical Research of Hunan Normal University (No. 2020-204). The assay was performed from 09:00 am to 11:00 a.m. or 16:00 p.m. to 18:00 p.m. by two volunteers standing at each monitoring point for 15 min separately. All the mosquitoes that landed and probed on volunteers’ skin and clothes were captured by mosquito aspirators. After morphological identification at species level and sex determination of all the collected mosquitoes^11^, the number of female *A. albopictus* were recorded by collection time, date and location.

### Genetic diversity analysis of *A. albopictus*

The gDNA was extracted from randomly selected factory-produced HC males and females as well as females and males collected from the control and release sites each month (June 2020 to October 2020) and then used for mitochondrial *COI* and *ND5* gene amplification on a Bio-Rad T100 thermal cycler. Using GoTaq Green Master Mix (Promega M7122), mitochondrial *COI* gene was amplified with specific primers LCO1490 and HCO2198^36^ as follows: initial denaturation at 95 °C for 3 min; a total of 35 cycles of denaturation at 95 °C for 30 s, annealing at 45 °C for 50 s, and extension at 72 °C for 1 min; and a final extension at 72 °C for 10 min. Mitochondrial *ND5* gene was detected by primers ND5F (5’-TCCTTAGAATAAAATCCCGC-3′) and ND5R (5′-GTTTCTGCTTTAGTTCATTCTTC-3′)^37^ as follows: initial denaturation at 95 °C for 3 min; a total of 35 cycles of denaturation at 95 °C for 30 s, annealing at 50 °C for 50 s, and extension at 72 °C for 1 min; and a final extension at 72 °C for 10 min. Then, the PCR products were purified using a QuiMag Gel Micro PCR Products Purification Kit (Sangon, B518747) and subjected to paired-end sequencing on an ABI 3730XL DNA analyzer at Sangon Biotech (Shanghai) Co., Ltd.

The sequenced data were subjected to phylogenetic analysis using MEGAX software v10.0.8^38^ followed by multiple sequence alignment by ClustalX 2.1^39^. The reference *COI* sequences of *A. albopictus* belonging to 25 haplotypes (GenBank accession numbers: KY378914-KY378938) identified in China, 12 *ND5* sequences of *A. albopictus* including the haplotype 1–8 reference sequences (GenBank accession numbers: KU926306, KU926308, and KX784180- KX784184) and representative sequences from Hunan and Guangdong Province of China (GenBank accession numbers: MF752529, MF752515, and MF752588-MF752589) were downloaded from GenBank^40^. Using *COI* of *Culex tritaeniorhynchus* (GenBank accession number: KJ012246.1) or *ND5* of *Anopheles arabiensis* (GenBank accession number: KJ950302.1) as an outgroup, maximum likelihood (ML) phylogenetic trees were established for *COI* and *ND5* genes, respectively, based on the general time reversible (GTR) genetic distance and nucleotide substitution model GTR + I + G with 1000 bootstraps to test the reliability of the branches.

### Statistics and reproducibility

GraphPad Prism 9.0 and IBM SPSS statistic 25.0 software were used for the statistical analysis. The two-sided Mann–Whitney U test was employed for the comparison of the median of weekly CIS index between release phase I (n = 5, 5 weeks monitoring for weekly CIS index) and release phase II (*n* = 8, 8 weeks monitoring for weekly CIS index, the weekly CIS index at thirteenth week was excluded as described above in the assay of HC male mating competitiveness in the field), the difference of weekly number of *w*Pip-positive samples from release and buffer sites between phase I (females: *n* = 5, larvae: *n* = 5, 5 weeks detection for weekly number of *w*Pip-positive samples) and phase II (females: *n* = 9, larvae: *n* = 9, 9 weeks detection for weekly number of *w*Pip-positive samples), the differential analysis between the data from the release and control sites within each stage, including the number of hatched eggs per trap (pre-release phase: release site: *n* = 90, 4796 hatched eggs from 90 retrieved ovitraps, control site: *n* = 90, 3841 hatched eggs from 90 retrieved ovitraps; phase I: release site: *n* = 143, 5381 hatched eggs from 143 retrieved ovitraps, control site: *n* = 148, 8872 hatched eggs from 148 retrieved ovitraps; phase II: release site: *n* = 269, 1592 hatched eggs from 269 retrieved ovitraps, control site: n = 262, 9506 hatched eggs from 262 retrieved ovitraps; post-release phase-2020: release site: n = 90, 71 hatched eggs from 90 retrieved ovitraps, control site: *n* = 89, 2230 hatched eggs from 89 retrieved ovitraps; post-release phase-2021: release site: *n* = 147, 3121 hatched eggs from 147 retrieved ovitraps, control site: *n* = 150, 2888 hatched eggs from 150 retrieved ovitraps), the average number of hatched eggs per trap in the prerelease stage (release site: *n* = 3, control site: *n* = 3, 3 weeks monitoring for average number of hatched eggs per trap), the number of females per trap per 24 h (pre-release: release site: n = 30, 298 females collected from 30 retrieved BG traps, control site: *n* = 30, 200 females collected from retrieved 30 BG traps; phase I: release site: *n* = 25, 282 females collected from 25 retrieved BG traps, control site: *n* = 25, 427 females collected from 25 retrieved BG traps; phase II: release site: *n* = 45, 123 females collected from 45 retrieved BG traps, control site: *n* = 45, 533 females collected from 45 retrieved BG traps; post-release phase-2020: release site: *n* = 15, 21 females collected from 15 retrieved BG traps, control site: *n* = 15, 137 females collected from 15 retrieved BG traps; post-release phase-2021: release site: *n* = 25, 230 females collected from 25 retrieved BG traps, control site: *n* = 25, 333 females collected from 25 retrieved BG traps.), the average number of females per trap per 24 h in prerelease stage (release site: *n* = 6, control site: *n* = 6, 6 times monitoring for average number of hatched eggs per trap per 24 h), and the average percentage of egg hatching per ovitrap (pre-release: release site: *n* = 3, control site: *n* = 3, 3 weeks monitoring for egg hatching; phase-I: release site: *n* = 5, control site: *n* = 5, 5 weeks monitoring for egg hatching; phase-II: release site: *n* = 9, control site: *n* = 9, 9 weeks monitoring for egg hatching; post-release-2020: release site: *n* = 3, control site: *n* = 3, 3 weeks monitoring for egg hatching; post-release-2021, release site: *n* = 5, control site: *n* = 5, 5 months monitoring for egg hatching). Pearson’s correlation test was used to measure the correlation of precipitation with the average number of adult female *A. albopictus* at the control site (precipitation: *n* = 20, average number of adult female: *n* = 20, 20 weeks monitoring for average number of females per trap per 24 h) and release stie (precipitation: *n* = 18, average number of adult female: n = 18, 18 weeks monitoring for average number of females per trap per 24 h), respectively, as well as the relationship between the number of *A. albopictus* at the release site and the control area in the prerelease stage (June 21, 2020, to July 6, 2020), based on the average number of hatched eggs per trap (release site: *n* = 3, control site: *n* = 3, 3 weeks monitoring for average number of hatched eggs per trap) and average number of females per trap (release site: *n* = 6, control site: *n* = 6, 6 weeks monitoring for weekly average number of hatched eggs per trap). A two-sided paired t test was undertaken to measure the variation in biting rate at the control and release sites in before-release stage (from June 21, 2020, to July 6, 2020, release stie: *n* = 4, control site: *n* = 4, 4 times human landing assay for biting rate.) and after-release stage (from July 6, 2020, to November 1, 2020, release stie: *n* = 7, control site: *n* = 7, 7 times human landing assay for biting rate).

### Reporting summary

Further information on research design is available in the Nature Portfolio Reporting Summary linked to this article.

## Supplementary information

Supplementary Information

Description of Additional Supplementary Files

Supplementary Data 1

Reporting Summary

## Data Availability

All data are available in the main text or in the supplementary materials. The numerical source data for all applicable graphs is provided in the excel file named “Supplementary Data 1”. All sequencing data have been submitted to GenBank with the accession numbers OP897744-OP897760 for mitochondrial *ND5* genes, and OP899376-OP899392 for *COI* genes. All other information is available from the corresponding authors upon reasonable request.
