# Supplementary material for: A standalone incompatible insect technique enables mosquito suppression in the urban subtropics
Source: Commun Biol. 2022 Dec 27;5:1419. doi: 10.1038/s42003-022-04332-6 (PMC9793375; doi:10.1038/s42003-022-04332-6)
Supplement: Supplementary file 1 — Supplementary Information [file 42003_2022_4332_MOESM1_ESM.pdf]

Supplementary figures:

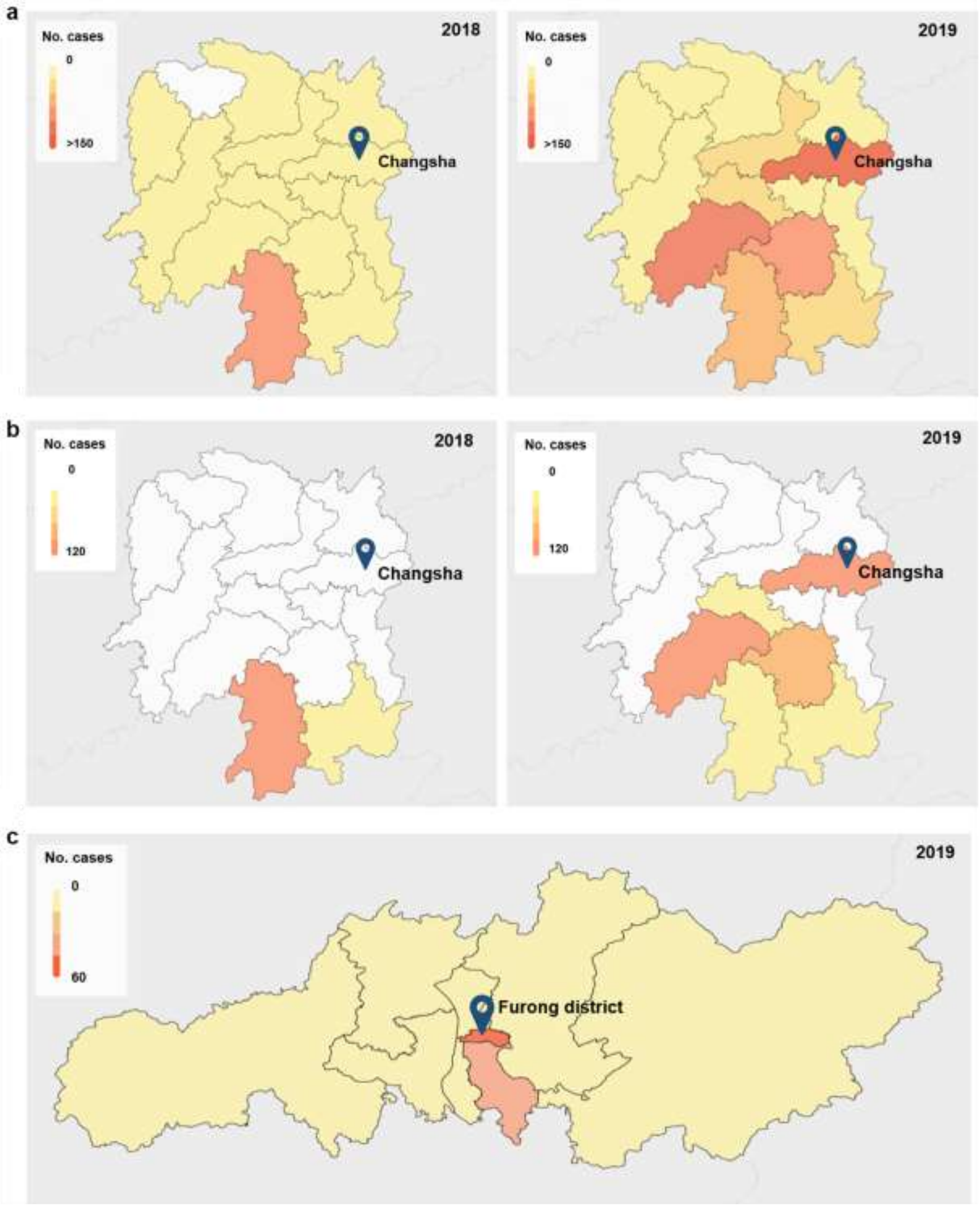

Supplementary Fig. 1. The distribution of dengue cases in Changsha, Hunan, China. a The distribution of total dengue cases in Hunan Province in 2018 and 2019. **b** The distribution of locally acquired dengue cases in Hunan

Province in 2018 and 2019. **c** The distribution of locally acquired dengue cases in Changsha in 2019. These dengue case data were downloaded from the Chinese National Notifiable Infectious Disease Reporting Information System (CNNDS) (http://www.chinacdc.cn/).

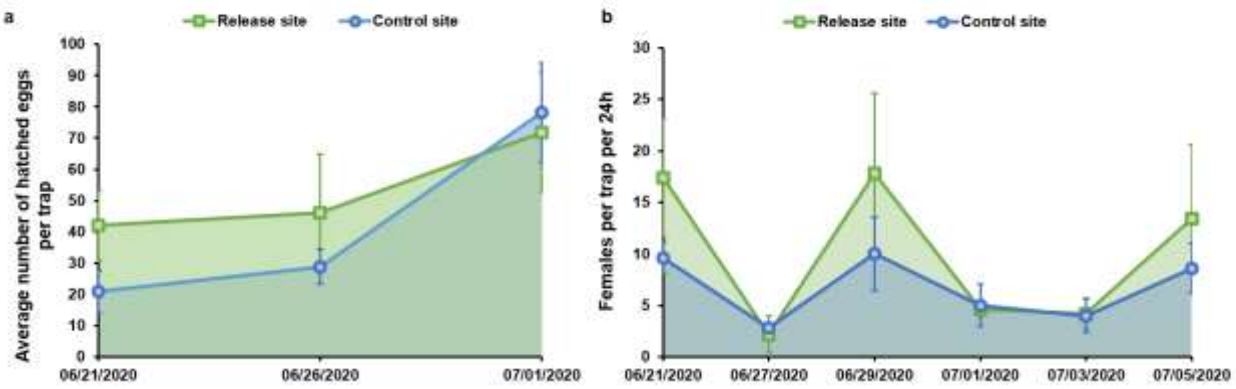

Supplementary Fig. 2. The mean number of hatched eggs and adult females (trap^{-1} week^{-1}) in the release and control sites during the prerelease phase. a The average number of hatched *A. albopictus* eggs in the release (green line) and control (blue line) sites during the prerelease stage. Pearson correlation: r=1, release site: n=3, control site: n=3, *P*=0.0028; two-sided Mann–Whitney test: release site: n=3, control site: n=3, *P*=0.7. **b** The average number of females collected weekly at the release (green line) and control (blue line) sites during the prerelease stage. Pearson correlation: r=0.9896, release site: n=6, control site: n=6, *P*=0.0002; two-sided Mann–Whitney test: release site: n=6, control site: n=6, *P*=0.5887. The date on x-axis is the starting date of weekly monitoring via ovitraps (a) and BG traps (b). The error bars present the standard error of mean.

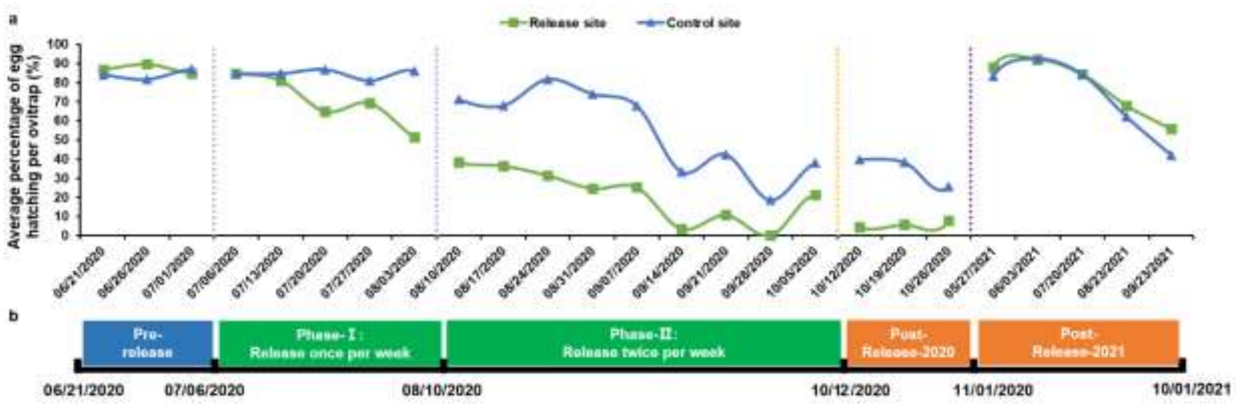

Supplementary Fig. 3. The egg hatch rates before, during and after release. a The weekly average percentage of egg hatching per ovitrap at the release (green line) and control (blue line) sites. The vertical dotted lines indicate the interval of each release phase. The date on x-axis is the starting date of weekly monitoring via ovitraps. Two-sided Mann–Whitney test: Pre-release: release site: n = 3, control site: n = 3, *P* = 0.4000; Phase-I: release site: n=5, control site: n=5, *P*=0.0317; Phase-II: release site: n = 9, control site: n = 9, *P* = 0.0040; Post-release-2020: release site: n = 3, control site: n=3, *P* = 0.1000; Post-release-2021: release site: n = 5, control site: n = 5, *P* = 0.6905. **b** Release schedule.

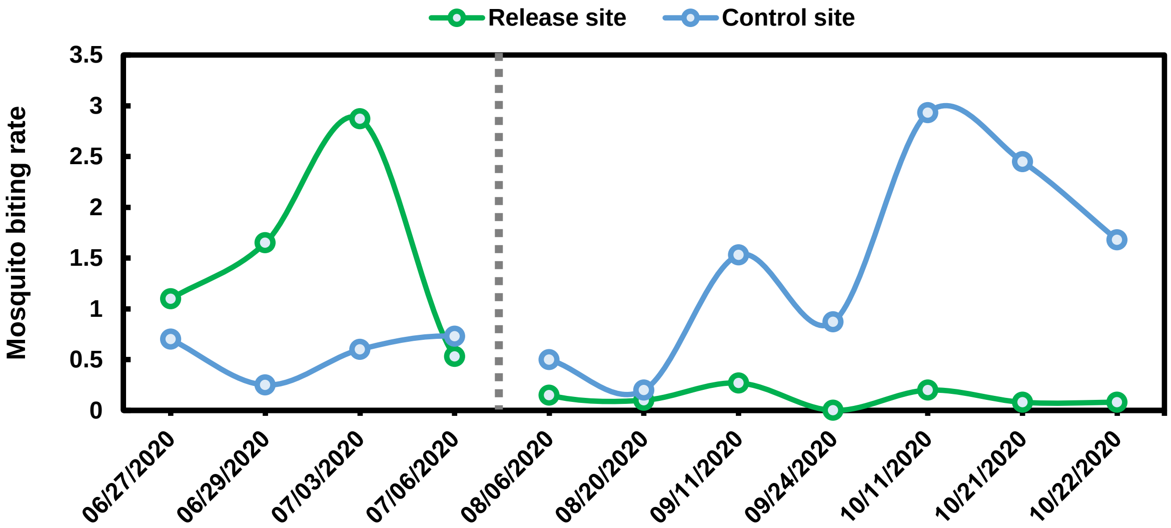

Supplementary Fig. 4. The mosquito biting rates at the release and control sites. Mosquito human landing catches in the release and control sites. The vertical dotted grey line indicates the start of release. Two-sided paired t test, June 27, 2020 to July 6, 2020 (Before-release): t=1.778, release site: n=4, control site: n=4, 3 degrees of freedom, *P*=0.1734; August 6, 2020 to October 22, 2020 (After-release): t=3.565, release site: n=7, control site: n=7, 6 degrees of freedom, *P*=0.0119.
