# Supplementary material for: A standalone incompatible insect technique enables mosquito suppression in the urban subtropics
Source: Commun Biol. 2022 Dec 27;5:1419. doi: 10.1038/s42003-022-04332-6 (PMC9793375; doi:10.1038/s42003-022-04332-6)
Supplement: Supplementary file 2 — Description of Additional Supplementary Files [file 42003_2022_4332_MOESM2_ESM.pdf]

Description of Additional Supplementary Files

File name: Supplementary Data 1

Description: numerical source data underlying graphs in excel format.
