# Supplementary material for: A standalone incompatible insect technique enables mosquito suppression in the urban subtropics
Source: Commun Biol. 2022 Dec 27;5:1419. doi: 10.1038/s42003-022-04332-6 (PMC9793375; doi:10.1038/s42003-022-04332-6)
Supplement: Supplementary file 4 — Reporting Summary [file 42003_2022_4332_MOESM4_ESM.pdf]

nature portfolio

Corresponding author(s): Xiaoling Pan and Zhiyong Xi

Last updated by author(s): Nov 30, 2022

Reporting Summary

Nature Portfolio wishes to improve the reproducibility of the work that we publish. This form provides structure for consistency and transparency in reporting. For further information on Nature Portfolio policies, see our Editorial Policies and the Editorial Policy Checklist.

Statistics

For all statistical analyses, confirm that the following items are present in the figure legend, table legend, main text, or Methods section.

n/a	Confirmed	
☐	☒	The exact sample size (*n*) for each experimental group/condition, given as a discrete number and unit of measurement
☐	☒	A statement on whether measurements were taken from distinct samples or whether the same sample was measured repeatedly
☐	☒	The statistical test(s) used AND whether they are one- or two-sided<br>*Only common tests should be described solely by name; describe more complex techniques in the Methods section.*
☐	☒	A description of all covariates tested
☐	☒	A description of any assumptions or corrections, such as tests of normality and adjustment for multiple comparisons
☐	☒	A full description of the statistical parameters including central tendency (e.g. means) or other basic estimates (e.g. regression coefficient) AND variation (e.g. standard deviation) or associated estimates of uncertainty (e.g. confidence intervals)
☐	☒	For null hypothesis testing, the test statistic (e.g. *F*, *t*, *r*) with confidence intervals, effect sizes, degrees of freedom and *P* value noted<br>*Give P values as exact values whenever suitable.*
☒	☐	For Bayesian analysis, information on the choice of priors and Markov chain Monte Carlo settings
☒	☐	For hierarchical and complex designs, identification of the appropriate level for tests and full reporting of outcomes
☐	☒	Estimates of effect sizes (e.g. Cohen's *d*, Pearson's *r*), indicating how they were calculated

Our web collection on statistics for biologists contains articles on many of the points above.

Software and code

Policy information about availability of computer code

Data collection	No software was used.
Data analysis	Maps and satellite images was analyzed with Bigemap v30.0.0.0 software (www.bigemap.com). The heatmap acquisition was performed using Map Lab (https://maplab.amap.com). The sequenced data were subject to phylogenetic analysis using MEGAX software v10.0.8 followed by multiple sequence alignment by Clustal 2.1. GraphPad Prism 9 and IBM SPSS statistic 25.0 software were used for statistical analysis.

For manuscripts utilizing custom algorithms or software that are central to the research but not yet described in published literature, software must be made available to editors and reviewers. We strongly encourage code deposition in a community repository (e.g. GitHub). See the Nature Portfolio guidelines for submitting code & software for further information.

Data

Policy information about availability of data

All manuscripts must include a data availability statement. This statement should provide the following information, where applicable:

- Accession codes, unique identifiers, or web links for publicly available datasets
- A description of any restrictions on data availability
- For clinical datasets or third party data, please ensure that the statement adheres to our policy

All data are available in the main text or in the supplementary materials. The numerical source data for all applicable graphs is provided in the excel file named

"Supplementary Data 1". All sequencing data have been submitted to GenBank with the accession numbers OP897744-OP897760 for mitochondrial ND5 genes, and OP899376-OP899392 for COI genes. All other information is available from the corresponding authors upon reasonable request.

Human research participants

Policy information about studies involving human research participants and Sex and Gender in Research.

Reporting on sex and gender	There is no data generated from human participants. Participants for studies of mosquito human landing assay are research volunteers including male and female.
Population characteristics	Participants for studies of mosquito bite rate are research volunteers (>18 years old, both male and female, Asian) in the filed sites in Changsha. As the research is not involved in studies of outcome of disease and treatment, no information on diagnosis and treatment categories are collected.
Recruitment	The research volunteers without certain underlying medical conditions and not in the menstrual period were recruited to participate in mosquito bite rate studies. To avoid biases caused by odor or chemical component, research volunteers were not allowed to use skin care products and externally applied agents.
Ethics oversight	Ethics Committee on Biomedical Research of Hunan Normal University.

Note that full information on the approval of the study protocol must also be provided in the manuscript.

Field-specific reporting

Please select the one below that is the best fit for your research. If you are not sure, read the appropriate sections before making your selection.

☒ Life sciences ☐ Behavioural & social sciences ☐ Ecological, evolutionary & environmental sciences

For a reference copy of the document with all sections, see nature.com/documents/nr-reporting-summary-flat.pdf

Life sciences study design

All studies must disclose on these points even when the disclosure is negative.

Sample size	No sample-size calculations were performed. Sample size was determined to be adequate based on the magnitude and consistency of measurable differences between groups.
Data exclusions	On principle, data were excluded for failed experiments which included suboptimal activation, and microbial contamination.
Replication	Replicate experiments were successful.
Randomization	The investigators, volunteers and traps were allocated randomly into both experimental and control groups for sample collection. The wild mosquito samples collected from experimental and control groups were allocated randomly into different batches for sex determination and detection of Wolbachia wPip infection.
Blinding	Blinding was not possible during sample collection, sex determination and mosquito human landing assay as release site, buffer site and control site were known by investigators. The blinding was used in detection of Wolbachia wPip infection and genetic diversity analysis.

Reporting for specific materials, systems and methods

We require information from authors about some types of materials, experimental systems and methods used in many studies. Here, indicate whether each material, system or method listed is relevant to your study. If you are not sure if a list item applies to your research, read the appropriate section before selecting a response.

Materials & experimental systems

n/a	Involved in the study	
☒	☐	Antibodies
☒	☐	Eukaryotic cell lines
☒	☐	Palaeontology and archaeology
☒	☐	Animals and other organisms
☒	☐	Clinical data
☒	☐	Dual use research of concern

Methods

n/a	Involved in the study	
☒	☐	ChIP-seq
☒	☐	Flow cytometry
☒	☐	MRI-based neuroimaging
